# Fertility preservation and reproductive outcomes in young women with breast and gynecological cancers: a new challenge for the clinician

**DOI:** 10.3389/fonc.2026.1904867

**Published:** 2026-09-18

**Authors:** Giusi Santangelo, Noemi Tonti, Lucrezia Lamorgese, Tullio Golia D’Augé, Innocenza Palaia, Federica Tomao, Violante Di Donato, Giorgia Perniola, Ludovico Muzii

**Affiliations:** 1 Department of Gynecological, Obstetrical and Urological Sciences, “Sapienza” University of Rome, Rome, Italy; 2 Obstetrics and Gynecology Unit, Maternal and Child Department, San Pietro Fatebenefratelli Hospital, Rome, Italy; 3 Department of Translational Medicine, University of Ferrara, Ferrara, Italy

**Keywords:** breast cancer, cancer survivors, cryopreservation, fertility counseling, fertility preservation strategies, fertility sparing, gynecological cancer

## Abstract

The increasing survival rates among young women diagnosed with cancer have shifted attention toward long-term quality-of-life issues, particularly fertility preservation and reproductive outcomes. Advances in cancer therapies have significantly improved prognosis in breast and gynecological malignancies; however, chemotherapy, radiotherapy, and radical surgery may severely compromise ovarian function and reproductive potential, leading to premature ovarian failure, infertility, and important psychological consequences. This review aims to summarize the current evidence regarding fertility preservation strategies and reproductive outcomes in young women with breast and gynecological cancers. An extensive literature review was conducted through MEDLINE, Embase, PubMed, and Cochrane databases, selected studies focusing on fertility counseling, fertility-sparing treatments, cryopreservation techniques, and reproductive outcomes in young women affected by cancer. Current evidence highlights the importance of early reproductive counseling and a multidisciplinary approach involving oncologists, gynecologists, reproductive specialists, and psychologists. Counseling should address ovarian reserve assessment, treatment-related gonadotoxicity, available fertility preservation options, and future pregnancy perspectives. Several fertility preservation strategies are currently available, including gonadotropin-releasing hormone agonists, oocyte and embryo cryopreservation, ovarian tissue cryopreservation, and fertility-sparing surgical approaches. In breast cancer, these strategies have demonstrated encouraging reproductive outcomes without compromising oncologic safety. In gynecological malignancies, conservative treatments such as hormonal therapy for endometrial cancer, conization or trachelectomy for cervical cancer, and fertility-sparing surgery for selected ovarian cancers may preserve reproductive potential in carefully selected patients. Available data suggest that pregnancy after fertility-sparing treatment is generally feasible and safe, although an increased risk of adverse obstetric outcomes, including preterm birth and low birth weight, has been reported. Despite significant progress in reproductive medicine and conservative oncologic surgery, several challenges remain unresolved, including optimal timing for pregnancy after cancer treatment, long-term follow-up strategies, and patient selection criteria. Ongoing research and multidisciplinary collaboration are essential to optimize fertility preservation protocols and improve both oncologic and reproductive outcomes in young cancer survivors.

## 1 Introduction

Annually, Italy records approximately 11, 000 newly diagnosed cases of cancer in females under the age of 40. Moreover, the global incidence of gynecological cancers in individuals under 40 is estimated to be around 195, 000 cases per year (1, 2). The most common types of cancer in young women are breast cancer, thyroid cancer, melanoma, cervical cancer, endometrial cancer, ovarian cancer, leukemia/lymphomas, and colorectal cancer (3). Breast Cancer (BC) is the most common cancer in women under 49 (40% of all cancer cases). Despite the typical rise in BC incidence with advancing age, a notable 7–10% of diagnosed cases are identified in women under the age of 40. The incidence trend in Italy is slightly increasing (0.3% per year), whereas mortality continues to decline (−0.8% per year). Indeed, the 5-year survival rate in young women (15–44 years old) is 91% (4). In the last few decades, the growing success of cancer treatments has led to a considerable increase in the number of cancer survivors. Nevertheless, several therapeutic approaches exert deleterious effects on the ovaries, posing a substantial risk of premature ovarian failure and, consequently, infertility in these individuals. For that reason, understanding the impact of cancer therapies on reproductive health is crucial to provide comprehensive care to female cancer survivors. Fertility loss can cause devastating emotional reactions in women impacting their quality of life. This review synthesizes existing literature by examining studies, clinical trials, and relevant data sources pertaining to cancer survivorship and fertility in women, with the aim to explore the intricate relationship between cancer survivorship and its implications for fertility.

## 2 Materials and methods

In January 2026, different authors conducted an extensive literature search to identify pertinent trials across various databases (MEDLINE, Embase, Pubmed, and Cochrane). The search strategy included combinations of controlled vocabulary (MeSH terms, where applicable) and free-text terms related to cancer and fertility preservation, including “Neoplasms”, “Cancer Survivors”, “Fertility Preservation”, “Fertility, “ “Infertility, Female”, “Cryopreservation”, “Oocyte Cryopreservation”, “Embryo Cryopreservation”, “Ovary”, “Gonadotropin-Releasing Hormone”, “Pregnancy”, “Breast Neoplasms”, and “Genital Neoplasms, Female”. These terms were combined using the Boolean operators AND and OR according to the specific database. The search focused on studies evaluating fertility counseling, treatment-related gonadotoxicity, fertility preservation strategies, fertility-sparing oncological treatments, and reproductive and pregnancy outcomes after cancer treatment. Only articles published in English were considered, with no predefined publication-year restriction. Priority was given to clinical guidelines, systematic reviews and meta-analyses, prospective and retrospective cohort studies, and relevant clinical trials. Letters, editorials, and case reports were excluded. Reference lists of relevant articles and international clinical guidelines were also screened to identify additional pertinent publications. Given the narrative nature of this review, study selection was guided by relevance to the predefined topics rather than by a formal systematic-review protocol. The selected publications were critically reviewed by the authors, and disagreements regarding their relevance were resolved by consensus (**Figure 1**).

**Figure 1:**
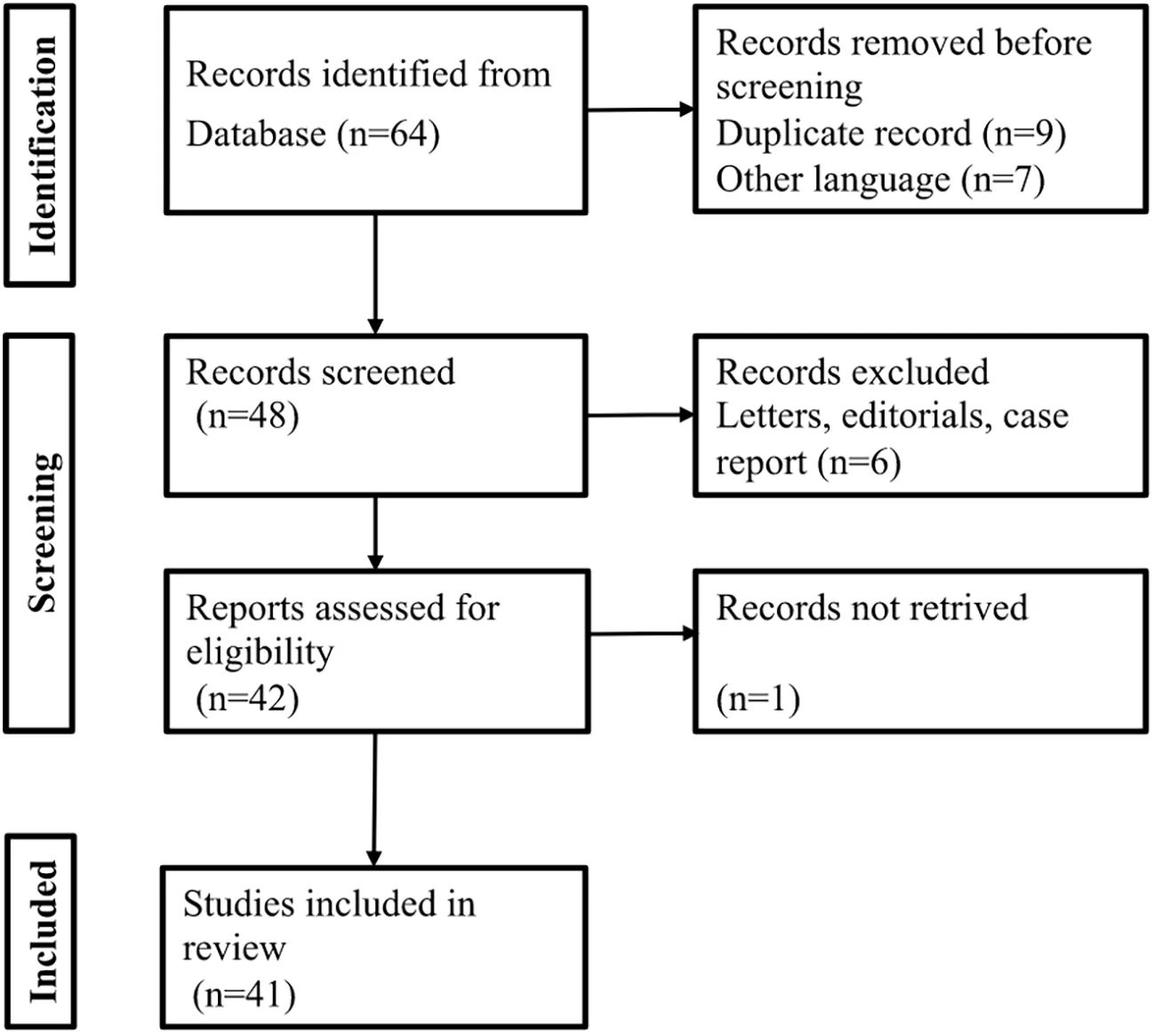
Flow diagram of search and selection of articles according the iclusion criteria.

## 3 Reproductive counseling in young woman with a diagnosis of cancer

Chemotherapy and radiotherapy remain the mainstay of cancer treatment. Both can be damaging to the ovary depending on the agent used, dose given, and age of the patient. These treatments are directly related to premature menopause, fertility loss and physical and psychological sequelae. Understanding these impacts is crucial for tailoring effective reproductive counseling strategies. Additionally, it is essential to consider not only the physical health but also the social and mental well-being of women undergoing a cancer diagnosis. Indeed, a study reported in the 2009 issue of Cancer indicated that couples dealing with a diagnosis of neoplasia experienced an approximately 11% rate of separations and divorces. Significantly, when the woman was the affected, this rate escalated to six times higher than the overall average (5). Fertility preservation is becoming crucial to improve the quality of life in cancer survivors. Despite national guidelines recommend the discussion of fertility preservation before initiating cancer therapies (6), there is a notable lack of implementation in this regard. Unfortunately, fertility preservation services are rarely offered or even discussed with the patient before treatment. Nevertheless, studies have shown that infertility is a significant survival concern. Indeed, patients who received information regarding their sexual and reproductive health had lower levels of psychological distress than patients who did not receive this information. Moreover, Informed decision reduces reproductive regret in these young men and women (7). Following the ASCO Clinical Practice Guidelines (8) clinicians should discuss with women who express an interest in fertility preservation several important issues. Indeed, counseling should involve a multidisciplinary approach whose objectives are to assess the pretreatment ovarian reserve, propose the most appropriate fertility preservation procedure, propose the method to minimize the gonadal toxicity, balance risks and benefits of choosing a treatment, and finally discuss the future probabilities of pregnancy. The clinician should discuss the feasibility of pursuing fertility preservation options based on recurrence risk and prognosis individualized for each patient. Moreover, it is necessary to discuss with the patient on the management of the toxicity of the treatment and on the management of the long-term collateral effects of the therapy. Finally, to carry out a correct counseling it is necessary to undertake an oncological-psychosocial rehabilitation and reintegration into the workplace (**Table 1**).

**Table 1:** Key communication strategies and goals in reproductive counseling for young cancer patients.

Counseling phase	Clinician’s objective	Key clinical message & tools
Initial Assessment	To evaluate the patient’s future reproductive desires and goals before initiating any gonadotoxic oncology treatments.	Implement validated screening questions; document the patient’s baseline ovarian reserve status and parity status.
Risk Information	To clearly communicate the potential short- and long-term impact of chemotherapy, radiotherapy, or surgery on fertility.	Provide standardized patient decision aids, educational brochures, and specific institutional oncofertility resources.
Decision Support	To minimize the patient’s decisional conflict and distress during an acute, highly stressful diagnostic phase.	Foster a shared decision-making (*SDM*) approach; address misconceptions regarding the oncological safety of fertility preservation.
Multidisciplinary Referral	To ensure timely access to specialized reproductive care without causing detrimental delays in cancer treatment.	Execute an immediate, urgent referral (within 24–48 hours) to a Reproductive Endocrinology and Infertility (*REI*) specialist.

REI, Reproductive Endocrinology and Infertility; SDM, Shared Decision-Making.

### 3.1 Available fertility preservation strategies

Several fertility preservation strategies are currently available, and their selection should be individualized according to the patient’s age, ovarian reserve, cancer type, gonadotoxicity of the planned treatment, and time available before therapy (8–10). Embryo and oocyte cryopreservation are established options for postpubertal women and should ideally be performed before starting gonadotoxic treatment; random-start ovarian stimulation protocols can minimize treatment delays. Ovarian tissue cryopreservation may be particularly useful when treatment cannot be postponed or in prepubertal patients, although careful selection is required because of the potential risk of reintroducing malignant cells. GnRH agonists administered during chemotherapy may reduce the risk of premature ovarian insufficiency, particularly in patients with breast cancer, but should not replace established cryopreservation techniques when these are feasible (11). Ovarian transposition may also be considered in selected patients undergoing pelvic radiotherapy to reduce ovarian radiation exposure (12). Overall, early referral to reproductive specialists and individualized multidisciplinary counseling are essential to select the most appropriate strategy and optimize future reproductive potential.

## 4 Damage from anti-cancer treatment: effects of chemotherapy and radiotherapy

The damage mediated by chemotherapy to fertility depends on patient’s age, ovarian reserve, type of chemotherapy and dosage (cumulative dose effect) (13). A study published in the Journal of Clinical Oncology in 2006 (14) revealed that among individuals with primary breast cancer undergoing chemotherapy, the likelihood of experiencing long-term amenorrhea in patients under 35 years old was comparable to that of women not undergoing chemotherapy. However, for patients aged 35 to 40 years, the risk of amenorrhea increased to 50%-85%, and for those over 40 years old, the risk escalated to 85%. Additionally, the study indicated that factors such as age, the chemotherapy regimen administered, and the use of tamoxifen influenced ovarian function. Many studies have investigated the type of damage of each chemotherapeutic agent on different cell types of the ovary. These findings have reported that ovarian damage can occur via several mechanisms (15). Chemotherapeutic agents can act at the level of the antral follicles, reducing their number, altering vascularization, and promoting a condition of fibrosis that results in follicular atresia. Concerning the chemotherapy agents employed, alkylating agents pose the greatest risk of inducing ovarian failure compared to other cytotoxic agents. Indeed, alkylators function by modifying DNA base pairs, resulting in cross-linking and the introduction of single-strand DNA breaks. Cyclophosphamide, Ifosfamide and Chlorambucil had the higher risk for ovaries, while agents such as Cisplatin, Carboplatin, Doxorubicin are considered at medium risk, finally, antimetabolite agents (Methotrexate, Fluorouracil) and antimicrotubule agents (Vincristine, Vinblastine) are considered low risk for ovaries damage (16) (**Table 2**).

**Table 2:** Risk stratification and pathogenetic mechanisms of chemotherapy-induced gonadotoxicity.

Gonadotoxic risk level	Chemotherapy classes & agents	Primary molecular & cellular mechanisms	Clinical reproductive outcomes
HIGH RISK *(>80% risk of permanent amenorrhea/POI)*	Alkylating Agents: • Cyclophosphamide • Ifosfamide • Chlorambucil	Cell-cycle non-specific DNA cross-linking. Triggers massive apoptosis of both resting primordial and growing follicles. Induces severe ovarian cortical fibrosis and microvascular damage.	High incidence of acute ovarian failure (AOF). Permanent Premature Ovarian Insufficiency (POI). Severe and irreversible depletion of the ovarian reserve.
INTERMEDIATE RISK *(30–70% risk of ovarian dysfunction)*	Platinum-Based Compounds: • Cisplatin • Carboplatin Anthracyclines: • Doxorubicin	Platinums: Form DNA adducts leading to double-strand breaks in oocytes and granulosa cells. Anthracyclines: Intercalate DNA and inhibit topoisomerase II; induce significant stromal and microvascular injury.	Temporary amenorrhea followed by variable partial recovery. Dose-dependent follicle loss leading to an accelerated onset of early menopause.
LOW/MINIMAL RISK *(<20% risk of long-term infertility)*	Antimetabolites: • Methotrexate • Fluorouracil (5-FU)	Cell-cycle specific (S-phase) inhibition of DNA synthesis. Primarily targets rapidly dividing granulosa cells of growing follicles, leaving the quiescent primordial follicle pool largely spared.	Temporary amenorrhea or menstrual irregularities during treatment. High probability of spontaneous resumption of normal menses and long-term fertility.

5-FU, Fluorouracil; AOF, Acute Ovarian Failure; POI, Premature Ovarian Insufficiency.

Radiotherapy may impair reproductive function through distinct effects on the ovaries and uterus. Ovarian follicles are highly radiosensitive, and clinically relevant follicular depletion may occur even at doses below 5 Gy (17). The extent of ovarian damage depends on the total radiation dose, fractionation, treatment field, and, importantly, the patient’s age and baseline ovarian reserve, with the radiation dose associated with permanent ovarian failure decreasing with increasing age (18). Ovarian exposure may result from direct irradiation or scattered radiation when the ovaries are located near the treatment field. Uterine irradiation should be considered separately from ovarian toxicity. Radiation-induced vascular and myometrial damage may impair uterine growth, vascularization, and distensibility, particularly when exposure occurs at a young age. These changes may persist even when ovarian function is preserved and are associated with adverse reproductive and obstetric outcomes, including miscarriage, fetal growth restriction, low birth weight, and preterm birth (19).

### 4.1 Reproductive effects of novel anticancer therapies

The increasing use of novel anticancer therapies has raised additional concerns regarding their potential impact on female reproductive function. These agents include immune checkpoint inhibitors, antibody-drug conjugates (ADCs), PARP inhibitors, CDK4/6 inhibitors, tyrosine kinase inhibitors, and other molecularly targeted therapies (20, 21). Unlike conventional cytotoxic chemotherapy, their gonadotoxic potential remains poorly characterized, as prospective studies evaluating ovarian reserve, premature ovarian insufficiency, and long-term reproductive outcomes are limited (22). Preclinical evidence and emerging clinical data suggest that some of these agents may interfere with ovarian function either directly or indirectly through pathways involved in follicular development, DNA repair, angiogenesis, or endocrine regulation. Immune checkpoint inhibitors may also affect reproductive function through immune-mediated endocrine toxicity (20, 21). However, the magnitude and reversibility of these effects remain uncertain. Therefore, patients of reproductive age receiving novel systemic therapies should undergo individualized fertility counseling before treatment, while further prospective studies are needed to define the gonadotoxic risk of these agents and their long-term impact on fertility and pregnancy outcomes.

## 5 The role of GnRH agonists in ovarian function preservation during chemotherapy

Gonadotropin-Releasing Hormone agonists (GnRHa) have emerged as a promising intervention to reduce the risk of POF by temporarily suppressing ovarian activity. GnRHa acts by desensitizing the GnRH receptors in the pituitary gland, leading to a reversible downregulation of the activity of hypothalamic-pituitary-gonadal axis resulting in decreased secretion of follicle-stimulating hormone (FSH) and luteinizing hormone (LH). Consequently, ovarian function is suppressed. By inducing a state of reversible ovarian quiescence, GnRHa reduces the metabolic and cellular activity of ovarian follicles, which may protect them from the cytotoxic effects of chemotherapy or radiation. The strongest evidence supporting their protective effect derives from premenopausal women with breast cancer receiving chemotherapy, in whom randomized trials and meta-analyses have shown a reduced incidence of chemotherapy-induced premature ovarian insufficiency and higher rates of ovarian-function recovery. Clinical studies have demonstrated the efficacy of GnRHa in preserving ovarian function and fertility in women undergoing gonadotoxic therapies (23). These studies have shown a reduction in the incidence of POF and improved ovarian function markers in patients treated with GnRHa compared to controls (23–25). Although the most robust and consistent evidence supporting the use of GnRHa derives from premenopausal women with breast cancer receiving chemotherapy, data are also available from studies including patients with other malignancies.

Del Mastro et al. (26) conducted a systematic review and meta-analysis of randomized trials evaluating the efficacy of GnRHa, administered before and during chemotherapy, in preventing POF in premenopausal cancer patients. Nine studies including 765 patients and 225 POF events were analyzed. POF occurred in 23% of patients receiving GnRHa compared with 37% of controls, supporting a protective effect of temporary ovarian suppression against chemotherapy-induced ovarian failure. Conversely, a randomized trial in patients with lymphoma failed to demonstrate a significant benefit of GnRHa in preventing chemotherapy-induced premature ovarian failure, while evidence in other malignancies remains limited and heterogeneous (27, 28),

Therefore, GnRHa should be considered an adjunctive strategy for ovarian-function preservation during chemotherapy rather than an alternative to established fertility preservation methods. Although GnRHa therapy offers potential benefits, it comes with limitations. Indeed, there is variability in individual response to GnRHa treatment, and the optimal duration and timing of administration are still on debate. For instance, further research is needed to elucidate the optimal protocols and patient selection criteria for GnRHa administration in the context of POF prevention (**Figure 2**).

**Figure 2:**
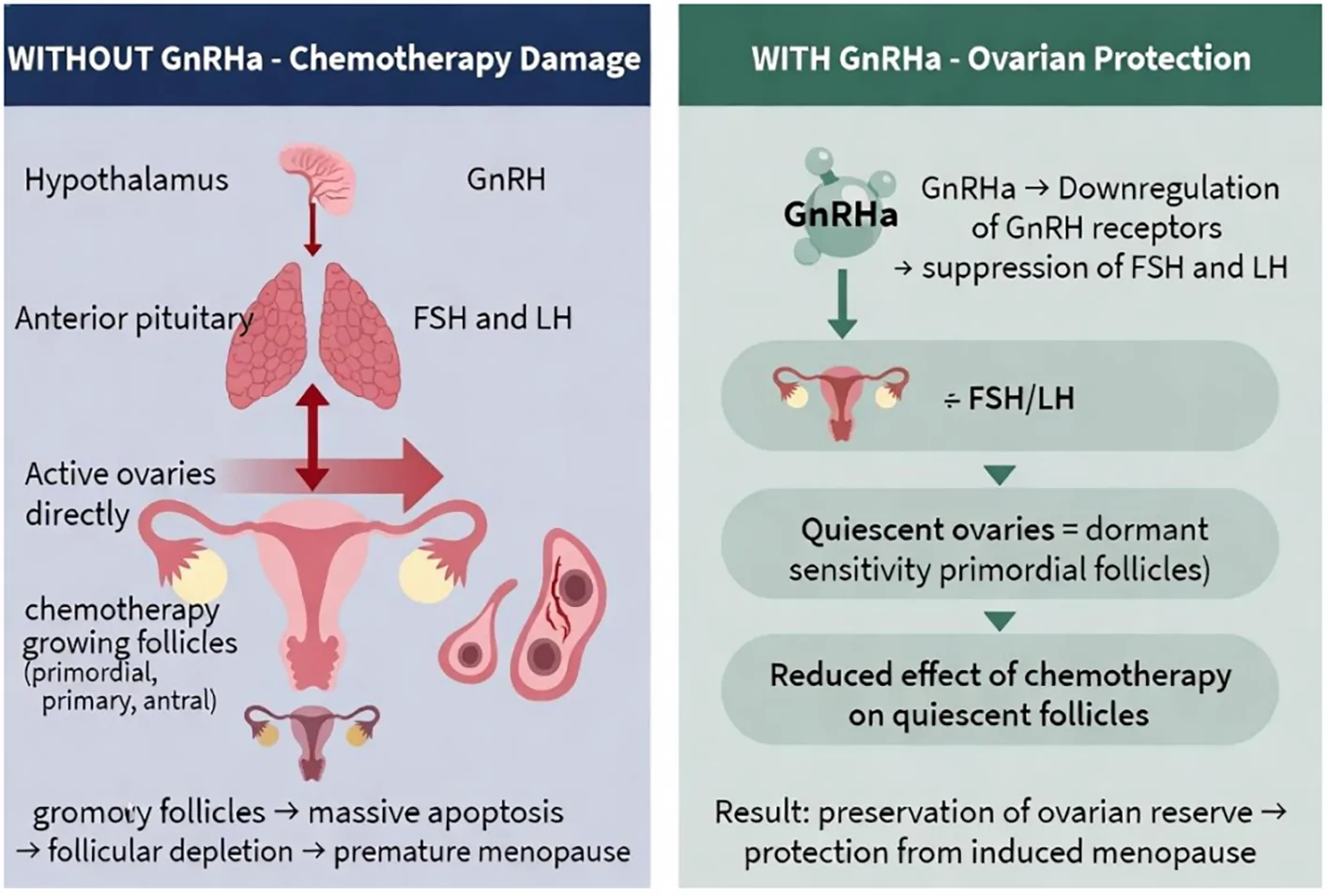
Mechanism of action of GnRHa in the prevention of chemotheraphy-induced menopause.

## 6 Outcomes of pregnancies after fertility-sparing treatment

Pregnancy outcomes after cancer treatment vary according to the underlying malignancy and treatment modality. Indeed, recent literature suggests a correlation between pregnancies following fertility-sparing treatment and various adverse outcomes. These include an increased incidence of abortions, complications during delivery, higher rates of cesarean sections, low birth weight, and an elevated risk of preterm birth (29–31). Systemic chemotherapy may primarily impair fertility through ovarian toxicity, whereas pelvic radiotherapy may additionally cause uterine vascular and myometrial damage, contributing to adverse obstetric outcomes even when ovarian function is preserved. Nevertheless, data regarding this topic are limited and still debated. In a retrospective study published in 2021 by Nitecki et al, the objective was to evaluate the outcomes of the first pregnancy after fertility-sparing surgery in patients treated for early-stage ovarian cancer. The primary outcome was preterm birth, and only the first pregnancy after cancer diagnosis was considered. Secondary outcomes included small-for-gestational-age (SGA) neonates, neonatal morbidity, and severe maternal morbidity. A total of 153 patients who conceived after fertility-sparing surgery were matched to 306 women in a control group. Finally, this study concluded that patients who conceived at least 3 months after surgery for early-stage ovarian cancer did not have an increased risk of adverse obstetric outcomes (32). In cervical cancer, reproductive and obstetric outcomes after fertility-sparing surgery are strongly procedure-dependent. Conization and simple trachelectomy are generally associated with more favorable reproductive outcomes than radical trachelectomy. A recent systematic review reported higher pregnancy and live-birth rates and a lower preterm birth rate following conization or simple trachelectomy compared with radical trachelectomy (18.3% vs. 33.3% for preterm birth) (33). Radical trachelectomy is also associated with an increased risk of premature rupture of membranes (34). In endometrial cancer, pregnancy is feasible after complete response to conservative treatment, with encouraging pregnancy and live-birth rates, particularly with assisted reproductive technologies (35). Overall, pregnancy counseling should be individualized according to cancer type, previous oncological treatments, and the type and extent of fertility-sparing surgery. For instance, based on these reassuring findings, more studies are needed to confirm these data to address pregnant women who underwent an oncological treatment to the correct management.

## 7 Fertility sparing treatments

### 7.1 Breast cancer

Fertility preservation is an essential consideration for young breast cancer patients facing gonadotoxic treatments. Current strategies such as ovarian suppression, oocyte and embryo cryopreservation, and ovarian tissue cryopreservation have shown promising results in preserving fertility and improving reproductive outcomes. Moreover, new strategies such as ovarian suppression with aromatase inhibitors or ovarian tissue transplantation are developing with the aim to contrast the limits of the previous methods. Nevertheless, safety of pregnancy in women with history of estrogen receptor (ER)-positive breast cancer remains controversial. In a multicenter case-control study, 333 patients with pregnancy after breast cancer were matched (1:3) to 874 nonpregnant patients of similar characteristics. At a median follow-up of 7.2 years after pregnancy, no difference in disease-free survival was observed between pregnant and nonpregnant patients with ER-positive (hazard ratio [HR] = 0.94, 95% confidence interval [CI] = 0.70 to 1.26, P = .68) or ER-negative disease (HR = 0.75, 95% CI = 0.53 to 1.06, P = .10). No overall survival (OS) difference was observed in ER-positive patients (HR = 0.84, 95% CI = 0.60 to 1.18, P = .32); ER-negative patients in the pregnant cohort had better OS (HR = 0.57, 95% CI = 0.36 to 0.90, P = .01). This study provides reassuring evidence on the long-term safety of pregnancy in breast cancer survivors, including those with ER-positive disease (36, 37). More recent evidence further supports these findings. A systematic review and meta-analysis showed no detrimental impact of subsequent pregnancy on disease-free or overall survival in women with hormone receptor-positive breast cancer (38). Reassuring data have also been reported in selected BRCA1/2 mutation carriers, with no significant adverse effect of subsequent pregnancy on oncological outcomes (39). Overall, these findings further support the oncological safety of pregnancy after breast cancer in appropriately selected patients. Although available evidence supports the oncological safety of pregnancy after breast cancer in appropriately selected patients, the most appropriate timing for conception remains a complex clinical issue. Data from Mueller et al. showed favorable survival outcomes among women who gave birth after breast cancer, particularly when childbirth occurred at least 10 months after diagnosis (40). However, pregnancy planning should take into account the individual clinical context, including previous and ongoing treatments, tumor characteristics, prognosis, reproductive age, and ovarian reserve. More recently, the POSITIVE trial provided reassuring prospective evidence in women with hormone receptor-positive early breast cancer who temporarily discontinued endocrine therapy to pursue pregnancy after receiving 18–30 months of treatment (37). These results support the feasibility of pregnancy in appropriately selected patients, while careful patient selection and continued long-term surveillance remain essential.

### 7.2 Endometrial cancer

Following the current guidelines (41) standard treatment for endometrial cancer, which involves total hysterectomy and bilateral salpingo-oophorectomy, has excellent survival outcomes, particularly for low-grade endometrioid tumors. However, it causes a permanent loss of fertility among women who wish to preserve their reproductive potential. Patients who can undergo fertility sparing treatment must be carefully selected. The criteria to select patients are:

1. Patients <40 aa.
2. Strong desire to be parents.
3. Endometrioid hystotype.
4. Well differentiated G1
5. Small tumor (without myometrial infiltration M </= 50%).
6. No synchronous/metacronous ovarian tumors.
7. No hereditary cancer syndrome.
8. No contraindication for HT.
9. Patients without comorbidities.

Fertility-sparing management of EC typically involves the use of the oral progestins MPA (Medroxyprogesterone acetate) or MA (megestrol acetate), or more recently the LNG-IUD (Levonorgestrel 52 mg). Current recommendations are MPA at a dose of 400–600 mg/day or MA at a dose of 160–320 mg/day for a minimum of 6 months, with follow-up assessment of treatment response using endometrial biopsy and imaging. Progestin use in women undergoing fertility-sparing treatment for atypical hyperplasia and EC have consistently shown good overall response rates (76–81%) (42). Moreover, another conservative fertility sparing technique is hysteroscopic tumor resection associated with hormonal therapy; Alonso et al. (43) reviewed studies published between 1975 and 2014 of EC patients aged <40 years treated with initial hysteroscopic resection followed by hormone therapy for fertility-sparing and found that the complete response rate for patients with FIGO stage 1A endometrioid grade 1 EC was 88.9%. Moreover, pregnancy rates among women who attempted conception, including through assisted reproductive technology (ART), ranged from 25% to 66%. This data suggests the potential superior outcomes of combined therapy compared to hormone treatment alone. Concerns have been raised about possible negative consequences of hysteroscopy due to EC cells spreading into the peritoneal cavity and the likelihood of adverse pregnancy outcomes due to mechanical damage to the basal layer of the endometrium causing Asherman’s syndrome and a risk of placenta accreta spectrum (44). Finding about these possible complications are still in doubt, for instance more studies are needed to confirm these data. In addition, is crucial that patients selected for fertility-sparing treatment understand and accept the need for close follow-up and endometrial sampling to evaluate the response to treatment. There isn’t a consensus among gynecologic oncology societies regarding the frequency of follow-up and the method of endometrial sampling, primarily because of differences in treatment protocols. Current recommendations suggest a histological evaluation at 3–6 months by endometrial biopsy. Establishment of a complete response, defined as no evidence of progressive disease, is a good indicator that pregnancy may be pursued, with ongoing close follow-up for early detection of successful pregnancy or signs of recurrence. Patients who partially respond to treatment at 6 months may be advised to continue progestin treatment for an additional 3–6 months, while non-responders at the 6-month follow-up with persistent disease confirmed by biopsy should be counselled to undergo the standard treatment (35). Moreover, Jeong-Yeol Park et al. in a study published in 2013 (45) analyzed pregnancy outcomes in young women with FIGO stage IA, grade 1 endometrioid adenocarcinoma of the uterus after successful fertility-sparing management using progestin. This study reviewed the medical records of 141 women who had complete remission after progestin treatment. Fifty-four (38.3%) women in the study cohort had a history of infertility. Seventy (49.6%) of the 141 patients tried to conceive with 44 (62.9%) receiving fertility drugs. The median interval to attempted pregnancy after treatment was 5 months (range 1–31 months). The median age at the time of the pregnancy trial was 32.4 years (range 23–40 years). Fifty-one (73%) of 70 women who tried to conceive were successful and 46 (66%) gave birth to 58 live neonates. The spontaneous abortion rate, ectopic pregnancy rate, and preterm delivery rates in our cohort were 24%, 2.8%, and 11.5%, respectively. The 5-year disease-free survival was similar between patients who received fertility drugs (n=44) or who did not (n=97) (73% compared with 62%, P = .335), and this rate was significantly higher in patients who achieved at least one pregnancy (n=51) than those who did not (n=90) (76% compared with 62%, P = .028). The conclusion is that although the proportion of patients with a history of subfertility or infertility was high, the pregnancy outcomes were very promising using assisted reproductive technology. Moreover, another interesting result is that the use of fertility drugs was not associated with a higher incidence of cancer recurrence after successful fertility-sparing management in this study population.

### 7.3 Cervical cancer

Following the current ESGO 2023 guidelines (46), fertility sparing treatment is a valid alternative for young patients with cervical cancer FIGO IA1-IB1 squamous cell carcinoma and HPV-related adenocarcinoma who want to preserve their fertility. Negative lymph nodal status is necessary for any fertility sparing therapy. Therefore, the ultra-staging of SLN is highly recommended and in case SLN cannot be detected a systematic pelvic lymphadenectomy should be performed. Moreover, free margins on final pathology are mandatory to considering fertility sparing management. The fertility sparing surgical treatments consist in sentinel or full pelvic lymphadenectomy, conization, simple/radical trachelectomy (abdominal, vaginal, laparoscopic, or robotic assisted). It is important to highlight that no randomized trial comparing these techniques has been published. Nevertheless, vaginal trachelectomy appears to have the best oncological and pregnancy outcome. The pregnancies outcomes in these patients have been investigated in several studies. Indeed, it has been seen that pregnancies following fertility sparing surgical have an increased risk of preterm delivery, PROM, lower birth weight and longer neonatal intensive care (47). These risk factors increase based on the type of surgical procedure underwent (low risk for conization, high risk for radical abdominal trachelectomy (48–51). For instance, in agreement with the following guidelines a permanent cerclage should be placed in all patients during simple or radical trachelectomy (46). The role of fertility sparing treatment in patients with tumors > 2 cm (FIGO IB2-3) is still debated due to the high risk of recurrence (51). Di Donato et al. conducted a systematic review (52) with the aim to investigated current evidence on oncological, fertility and obstetric outcomes of patients with stage I cervical cancer of 4 cm or larger undergoing fertility-sparing surgery. They considered fifteen studies involving 48 patients. Among these, three (6.3%) experienced a recurrence and one (2.1%) died of disease. The 5-year DFS rate was 92.4%. The 5-year OS rate was 97.6%. A significantly shorter 5-year DFS was reported for high-risk patients (G3, non-squamous histotype, diameter ≥5 cm) compared with low-risk (74.7% vs. 100%; log-rank test, p=0.024). Fertility outcomes data were available for 12 patients. Five patients (41.7%) attempted to conceive with an estimated pregnancy rate of 80%, a live birth rate of 83.3% and a premature delivery rate of 20%. For instance, this review concluded that women with high tumor grade G3, aggressive histology, and tumor size ≥5 cm have a higher risk of recurrence, while oncologic outcomes are encouraging among low-risk patients. A valid alternative in these patients has been found in NACT followed by fertility-sparing surgery, as demonstrated in several studies (53–55). However, the optimal number of chemotherapy cycles, the specific chemotherapy regimen, and the extent of cervical resection post- NACT have not yet been definitively determined (56) Follow up is the same as for any cervical cancer patient. While the optimal time to have a pregnancy after FSS is still an open question. Indeed, no guidance can be given regarding the earliest feasible timing for pursuing childbirth after fertility-sparing treatment.

### 7.4 Ovarian cancer

In select cases of early-stage ovarian cancer, fertility-preserving surgery, including unilateral oophorectomy or cystectomy, may be an option. This approach has the aim to preserve both ovarian function and fertility while ensuring effective cancer treatment. Bentivegna et al. in 2016 published a review to assess the fertility sparing treatment for ovarian cancer (57). This review comprises 1150 patients and 139 relapsing patients reported by 21 teams. Borderline ovarian tumors and non-EOCs were excluded. They concluded that conservative treatment can be safely carried out for stage IA and IC G1–2 disease and stage IC1 according to the new FIGO staging system. Nevertheless, the number of patients reported with grade 2 disease is too small to definitively confirm whether FSS is safe in this subgroup. For patients with ‘less favorable’ prognostic factors (grade 3 or stage IC3 disease), the safety of FSS could not be confirmed. FSS could probably be considered in stage I clear-cell tumors but should remain contraindicated for stage II/III disease (whatever the histologic subtype). For instance, as the disease stage and the histologic data (tumor type and grade) are crucial to patient selection for this treatment, this implies careful and mandatory complete surgical staging surgery in this context and a pathological analysis (or review) of the tumor by an expert pathologist.

## 8 Conclusion

In conclusion, fertility-sparing treatment plays a crucial role in addressing the reproductive needs of young women diagnosed with cancer. Current strategies offer valuable options for preserving fertility while effectively managing the disease. However, the available evidence and the effectiveness of these strategies vary according to cancer type and treatment modality. Patients who can undergo fertility sparing treatments must be strictly selected following the current guidelines. The clinician always needs to consider the type of tumor, the stage, the prognosis, and patient’s age at diagnosis. Achieving pregnancy after cancer treatment remains a challenge and requires individualized counseling based on cancer type, treatment received, and patient characteristics. Indeed, key unresolved issues include the optimal timing of pregnancy after fertility-sparing treatment and appropriate follow-up strategies. Moreover, evidence remains limited and heterogeneous in several clinical settings, highlighting the need for further prospective studies. Ongoing research and collaboration between oncologists, reproductive specialists, and patients are essential to optimize fertility-sparing treatment strategies and improve reproductive outcomes in this young population.

## References

[1] Sung H Ferlay J Siegel RL Laversanne M Soerjomataram I Jemal A . Global cancer statistics 2020: GLOBOCAN estimates of incidence and mortality worldwide for 36 cancers in 185 countries. CA Cancer J Clin. (2021) 71(3):209–249. doi: 10.3322/CAAC.21660 33538338

[2] I Numeri Del Cancro in Italia 2020 | Associazione Italiana Registri Tumori (2020). Available online at: https://www.registri-tumori.it/cms/pubblicazioni/i-numeri-del-cancro-italia-2020 (Accessed May 9, 2026).

[3] Mahajan N . Fertility preservation in female cancer patients: an overview. J Hum Reprod Sci. (2015) 8(1):3–12. Mumbai: Medknow Publications. doi: 10.4103/0974-1208.153119 PMC4381379 25838742

[4] Rossi L Mazzara C Pagani O . Diagnosis and treatment of breast cancer in young women. Curr Treat Options Oncol. (2018) 20(1):201–206. New York, NY: Springer. doi: 10.1007/s11864-019-0685-7 31776799

[5] Glantz MJ Chamberlain MC Liu Q Hsieh CC Edwards KR Van Horn A . Gender disparity in the rate of partner abandonment in patients with serious medical illness. Cancer. (2009) 115(22):5237–5242. doi: 10.1002/cncr.24577 19645027

[6] Linee Guida AIOM | AIOM (2020). Available online at: https://www.aiom.it/linee-guida-aiom/ (Accessed May 9, 2026).

[7] Quinn GP Vadaparampil ST Bell-Ellison BA Gwede CK Albrecht TL . Patient–physician communication barriers regarding fertility preservation among newly diagnosed cancer patients. Soc Sci Med. (2008) 66(3):784–789. doi: 10.1016/j.socscimed.2007.09.013 18023955

[8] Su HI Lacchetti C Letourneau J Partridge AH Qamar R Quinn GP . Fertility preservation in people with cancer: ASCO guideline update. J Clin Oncol. (2025) 43(12):1488–1515. doi: 10.1200/JCO-24-02782 40106739

[9] Anderson RA Amant F Braat D D'Angelo A De Sousa Lopes SMC Demeestere I . ESHRE guideline: female fertility preservation. Hum Reprod Open. (2021) 2020(4). doi: 10.1093/hropen/hoaa052 33225079 PMC7666361

[10] Gracia C Amato P Anderson J Flyckt R Hansen K Hill M . Fertility preservation in patients with medical indications: a committee opinion. Fertil Steril. (2026) 125(2):247–259. doi: 10.1016/J.FERTNSTERT.2025.12.001 41494900

[11] Lambertini M Moore HCF Leonard RCF Loibl S Munster P Bruzzone M . Gonadotropin-releasing hormone agonists during chemotherapy for preservation of ovarian function and fertility in premenopausal patients with early breast cancer: a systematic review and meta-analysis of individual patient-level data. J Clin Oncol. (2018) 36(19):1981–1990. doi: 10.1200/JCO.2018.78.0858 29718793 PMC6804855

[12] Klipstein S Abhari S Amato P Arjunan A Anderson D Bakare T . Fertility preservation and reproduction in patients facing gonadotoxic therapies or gonadectomy: an ethics committee opinion. Fertil Steril. (2026) 125(2):260–266. doi: 10.1016/J.FERTNSTERT.2025.12.002 41500852

[13] McLaren JF Bates GW . Fertility preservation in women of reproductive age with cancer. Am J Obstet Gynecol. (2012) 207(6):455–462. doi: 10.1016/J.AJOG.2012.08.013 22959764

[14] Petrek JA Naughton MJ Case LD Paskett ED Naftalis EZ Singletary SE . Incidence, time course, and determinants of menstrual bleeding after breast cancer treatment: a prospective study. J Clin Oncol. (2006) 24(7):1045–1051. doi: 10.1200/JCO.2005.03.3969 16476708

[15] Szymanska KJ Tan X Oktay K . Unraveling the mechanisms of chemotherapy-induced damage to human primordial follicle reserve: road to developing therapeutics for fertility preservation and reversing ovarian aging. Mol Hum Reprod. (2020) 26(8):553–566. Oxford: Oxford University Press. doi: 10.1093/MOLEHR/GAAA043 32514568 PMC7411370

[16] Epstein RJ . Drug-induced DNA damage and tumor chemosensitivity. J Clin Oncol. (1990) 8(12):2062–2084. doi: 10.1200/JCO.1990.8.12.2062 2230898

[17] Wallace WHB Thomson AB Kelsey TW . The radiosensitivity of the human oocyte. Hum Reprod. (2003) 18(1):117–121. doi: 10.1093/HUMREP/DEG016 12525451

[18] Wallace WHB Thomson AB Saran F Kelsey TW . Predicting age of ovarian failure after radiation to a field that includes the ovaries. Int J Radiat Oncol Biol Phys. (2005) 62(3):738–744. doi: 10.1016/J.IJROBP.2004.11.038 15936554

[19] Legrand C Schwartz B Bolle S Doz F Tsatsaris V Sarnacki S . Pregnancy outcomes in childhood cancer survivors according to uterine radiation dose and other factors. Br J Radiol. (2025) 98(1176):2079–2089. doi: 10.1093/BJR/TQAF130 40973203

[20] Helgadottir H Matikas A Fernebro J Frödin JE Ekman S Rodriguez-Wallberg KA . Fertility and reproductive concerns related to the new generation of cancer drugs and the clinical implication for young individuals undergoing treatments for solid tumors. Eur J Cancer. (2024) 202:114010. doi: 10.1016/J.EJCA.2024.114010 38520926

[21] Arecco L de Moura Leite L Gentile G Jankovic K Stana M Ottonello S . Gonadotoxicity of immunotherapy and targeted agents in patients with cancer and impact on subsequent pregnancies. Hum Reprod. (2025) 40(8):1452–1466. doi: 10.1093/HUMREP/DEAF096 40482082 PMC12314150

[22] Gentile G Scagnoli S Arecco L Santini D Botticelli A Lambertini M . Assessing risks and knowledge gaps on the impact of systemic therapies in early breast cancer on female fertility: a systematic review of the literature. Cancer Treat Rev. (2024) 128:102769. doi: 10.1016/J.CTRV.2024.102769 38810574

[23] Li ZY Dong YL Cao XZ Ren SS Zhang Z . Gonadotropin-releasing hormone agonists for ovarian protection during breast cancer chemotherapy: a systematic review and meta-analysis. Menopause. (2022) 29(9):1093. doi: 10.1097/GME.0000000000002019 35917530 PMC9422762

[24] Leonard RCF Adamson DJA Bertelli G Mansi J Yellowlees A Dunlop J . GnRH agonist for protection against ovarian toxicity during chemotherapy for early breast cancer: the Anglo Celtic Group OPTION trial. Ann Oncol. (2017) 28(8):1811–1816. doi: 10.1093/ANNONC/MDX184 28472240

[25] Del Mastro L Boni L Michelotti A Gamucci T Olmeo N Gori S . Effect of the gonadotropin-releasing hormone analogue triptorelin on the occurrence of chemotherapy-induced early menopause in premenopausal women with breast cancer: a randomized trial. JAMA. (2011) 306(3):269–276. doi: 10.1001/JAMA.2011.991 21771987

[26] Del Mastro L Ceppi M Poggio F Bighin C Peccatori F Demeestere I . Gonadotropin-releasing hormone analogues for the prevention of chemotherapy-induced premature ovarian failure in cancer women: systematic review and meta-analysis of randomized trials. Cancer Treat Rev. (2014) 40(5):675–683. London: W.B. Saunders Ltd. doi: 10.1016/j.ctrv.2013.12.001 24360817

[27] Demeestere I Brice P Peccatori FA Kentos A Dupuis J Zachee P . No evidence for the benefit of gonadotropin-releasing hormone agonist in preserving ovarian function and fertility in lymphoma survivors treated with chemotherapy: final long-term report of a prospective randomized trial. J Clin Oncol. (2016) 34(22):2568–2574. doi: 10.1200/JCO.2015.65.8864 27217453

[28] Demeestere I Brice P Peccatori FA Kentos A Gaillard I Zachee P . Gonadotropin-releasing hormone agonist for the prevention of chemotherapy-induced ovarian failure in patients with lymphoma: 1-year follow-up of a prospective randomized trial. J Clin Oncol. (2013) 31(7):903–909. doi: 10.1200/JCO.2012.42.8185 23129737

[29] Mogos MF Salihu HM Aliyu MH Whiteman VE Sultan DH . Association between reproductive cancer and fetal outcomes: a population-based study. Int J Gynecol Cancer. (2013) 23(2):218–226. doi: 10.1097/IGC.0B013E31827B877B 23314281

[30] Hartnett KP Ward KC Kramer MR Lash TL Mertens AC Spencer JB . The risk of preterm birth and growth restriction in pregnancy after cancer. Int J Cancer. (2017) 141(11):2187–2196. doi: 10.1002/IJC.30914 28836277 PMC5766343

[31] Stensheim H Klungsøyr K Skjærven R Grotmol T Fosså SD . Birth outcomes among offspring of adult cancer survivors: a population-based study. Int J Cancer. (2013) 133(11):2696–2705. doi: 10.1002/IJC.28292 23729011

[32] Nitecki R Clapp MA Fu S Lamiman K Melamed A Brady PC . Outcomes of the first pregnancy after fertility-sparing surgery for early-stage ovarian cancer. Obstet Gynecol. (2021) 137(6):1109–1118. doi: 10.1097/AOG.0000000000004394 33957660 PMC8141030

[33] Taliento C Scutiero G Battello G Sala A Pellecchia G Trozzi R . Reproductive, obstetrical and oncological outcomes of fertility-sparing treatment for cervical cancer according to the FIGO 2018 staging system: a systematic review. Eur J Surg Oncol. (2025) 51(1). doi: 10.1016/J.EJSO.2024.109359 39531915

[34] Nezhat C Roman RA Rambhatla A Nezhat F . Reproductive and oncologic outcomes after fertility-sparing surgery for early stage cervical cancer: a systematic review. Fertil Steril. (2020) 113(4):685–703. doi: 10.1016/J.FERTNSTERT.2020.02.003 32228873

[35] Rodolakis A Scambia G Planchamp F Acien M Di Spiezio Sardo A Farrugia M . ESGO/ESHRE/ESGE guidelines for the fertility-sparing treatment of patients with endometrial carcinoma. Int J Gynecol Cancer. (2023) 33(2):208–222. doi: 10.1136/IJGC-2022-004047 36746507

[36] Lambertini M Kroman N Ameye L Cordoba O Pinto A Benedetti G . Long-term safety of pregnancy following breast cancer according to estrogen receptor status. J Natl Cancer Inst. (2018) 110(4):426–429. doi: 10.1093/jnci/djx206 29087485 PMC6658852

[37] Partridge AH Niman SM Ruggeri M Peccatori FA Azim HA Colleoni M . Interrupting endocrine therapy to attempt pregnancy after breast cancer. N Engl J Med. (2023) 388(18):1645–1656. doi: 10.1056/NEJMOA2212856 37133584 PMC10358451

[38] Arecco L Blondeaux E Bruzzone M Latocca MM Mariamidze E Begijanashvili S . Safety of pregnancy after breast cancer in young women with hormone receptor-positive disease: a systematic review and meta-analysis. ESMO Open. (2023) 8(6). doi: 10.1016/J.ESMOOP.2023.102031 37879234 PMC10774870

[39] Perachino M Blondeaux E Delucchi V Bernstein Molho R Frank S Paluch-Shimon S . Safety of having a subsequent pregnancy after prior diagnosis of breast cancer during pregnancy in young BRCA carriers. ESMO Open. (2025) 10(8). doi: 10.1016/J.ESMOOP.2025.105513 40811881 PMC12362513

[40] Mueller BA Simon MS Deapen D Kamineni A Malone KE Daling JR . Childbearing and survival after breast carcinoma in young women. Cancer. (2003) 98(6):1131–1140. doi: 10.1002/cncr.11634 12973836

[41] Concin N Matias-Guiu X Cibula D Colombo N Creutzberg CL Ledermann J . ESGO–ESTRO–ESP guidelines for the management of patients with endometrial carcinoma: update 2025. Lancet Oncol. (2025) 26(8):e423–e435. doi: 10.1016/S1470-2045(25)00167-6 40744042

[42] Obermair A Baxter E Brennan DJ Mcalpine JN Mueller JJ Amant F . Fertility-sparing treatment in early endometrial cancer: current state and future strategies. Obstet Gynecol Sci. (2020) 63(4):417–431. doi: 10.5468/OGS.19169 32689770 PMC7393748

[43] Alonso S Castellanos T Lapuente F Chiva L . Hysteroscopic surgery for conservative management in endometrial cancer: a review of the literature. Ecancermedicalscience. (2015) 9. doi: 10.3332/ecancer.2015.505 25729418 PMC4335962

[44] Park JY . Hysteroscopy in fertility-sparing management for early endometrial cancer: a double-edged sword. J Gynecol Oncol. (2017) 28(1):e16. doi: 10.3802/jgo.2017.28.e16 27894168 PMC5165065

[45] Park JY Seong SJ Kim TJ Kim JW Kim SM Bae DS . Pregnancy outcomes after fertility-sparing management in young women with early endometrial cancer. Obstet Gynecol. (2013) 121(1):136–142. doi: 10.1097/AOG.0b013e31827a0643 23262938

[46] Cibula D Raspollini MR Planchamp F Centeno C Chargari C Felix A . ESGO/ESTRO/ESP guidelines for the management of patients with cervical cancer – update 2023. Virchows Arch. (2023) 482(6):935–966. doi: 10.1007/s00428-023-03552-3 37145263 PMC10247855

[47] Monti M D'Aniello D Scopelliti A Tibaldi V Santangelo G Colagiovanni V . Relationship between cervical excisional treatment for cervical intraepithelial neoplasia and obstetrical outcome. Minerva Obstet Gynecol. (2021) 73(2):233–246. doi: 10.23736/S2724-606X.20.04678-X 33140628

[48] Bjørge T Skare GB Bjørge L Tropé A Lönnberg S . Adverse pregnancy outcomes after treatment for cervical intraepithelial neoplasia. Obstet Gynecol. (2016) 128(6):1265–1273. doi: 10.1097/AOG.0000000000001777 27824756

[49] Kyrgiou M Athanasiou A Kalliala IEJ Paraskevaidi M Mitra A Martin-Hirsch PPL . Obstetric outcomes after conservative treatment for cervical intraepithelial lesions and early invasive disease. Cochrane Database Syst Rev. (2017) 11(11). doi: 10.1002/14651858.CD012847 29095502 PMC6486192

[50] Musella A Santangelo G Vertechy L Di Pinto A Sassu CM Fischetti M . Post-conization cervical stenosis treated with silicone catheter in microinvasive cervical cancer patient: a case report. Int J Surg Case Rep. (2020) 67:95–97. doi: 10.1016/j.ijscr.2020.02.005 32058308 PMC7013100

[51] Bogani G Lalli L Sopracordevole F Ciavattini A Ghelardi A Simoncini T . Development of a nomogram predicting the risk of persistence/recurrence of cervical dysplasia. Vaccines (Basel). (2022) 10(4). doi: 10.3390/vaccines10040579 35455328 PMC9029732

[52] Di Donato V Caruso G Sassu CM Santangelo G Bogani G Plotti F . Fertility-sparing surgery for women with stage I cervical cancer of 4 cm or larger: a systematic review. J Gynecol Oncol. (2021) 32(6). doi: 10.3802/jgo.2021.32.e83 34431254 PMC8550926

[53] Tesfai FM Kroep JR Gaarenstroom K De Kroon C Van Loenhout R Smit V . Fertility-sparing surgery of cervical cancer >2 cm (International Federation of Gynecology and Obstetrics 2009 stage IB1-IIA) after neoadjuvant chemotherapy. Int J Gynecol Cancer. (2020) 30(1):115–121. doi: 10.1136/IJGC-2019-000647 31744889

[54] Rendón GJ Lopez Blanco A Aragona A Saadi JM Di Guilmi J Arab Eblen C . Oncological and obstetrical outcomes after neo-adjuvant chemotherapy followed by fertility-sparing surgery in patients with cervical cancer ≥2 cm. Int J Gynecol Cancer. (2021) 31(3):462–467. doi: 10.1136/IJGC-2020-002076 33199429

[55] Kasius JC van der Velden J Denswil NP Tromp JM Mom CH . Neo-adjuvant chemotherapy in fertility-sparing cervical cancer treatment. Best Pract Res Clin Obstet Gynaecol. (2021) 75:82–100. doi: 10.1016/J.BPOBGYN.2021.01.010 33752979

[56] Perniola G Fischetti M Tomao F Di Donato V Palaia I Santangelo G . Evaluation of parametrial status in locally advanced cervical cancer patients after neoadjuvant chemotherapy: a prospective study on diagnostic accuracy of three-dimensional transvaginal ultrasound. Oncology. (2020) 98(9):603–611. Basel: S. Karger AG. doi: 10.1159/000506642 32492692

[57] Bentivegna E Gouy S Maulard A Pautier P Leary A Colombo N . Fertility-sparing surgery in epithelial ovarian cancer: a systematic review of oncological issues. Ann Oncol. (2016):1994–2004. doi: 10.1093/annonc/mdw311 27502723

